# High Content Screening Identifies Decaprenyl-Phosphoribose 2′ Epimerase as a Target for Intracellular Antimycobacterial Inhibitors

**DOI:** 10.1371/journal.ppat.1000645

**Published:** 2009-10-30

**Authors:** Thierry Christophe, Mary Jackson, Hee Kyoung Jeon, Denis Fenistein, Monica Contreras-Dominguez, Jaeseung Kim, Auguste Genovesio, Jean-Philippe Carralot, Fanny Ewann, Eun Hye Kim, Sae Yeon Lee, Sunhee Kang, Min Jung Seo, Eun Jung Park, Henrieta Škovierová, Ha Pham, Giovanna Riccardi, Ji Youn Nam, Laurent Marsollier, Marie Kempf, Marie-Laure Joly-Guillou, Taegwon Oh, Won Kyung Shin, Zaesung No, Ulf Nehrbass, Roland Brosch, Stewart T. Cole, Priscille Brodin

**Affiliations:** 1 Screening Technologies and Pharmacology, Institut Pasteur Korea, Bundang-gu, Seongnam-si, Gyeonggi-do, Korea; 2 Mycobacteria Research Laboratories, Department of Microbiology, Immunology and Pathology, Colorado State University, Fort Collins, Colorado, United States of America; 3 Image Mining, Institut Pasteur Korea, Bundang-gu, Seongnam-si, Gyeonggi-do, Korea; 4 Biology of Intracellular Pathogens Inserm Avenir Group, Institut Pasteur Korea, Bundang-gu, Seongnam-si, Gyeonggi-do, Korea; 5 Medicinal Chemistry, Institut Pasteur Korea, Bundang-gu, Seongnam-si, Gyeonggi-do, Korea; 6 Dipartimento di Genetica e Microbiologia, Università degli Studi di Pavia, Pavia, Italy; 7 Groupe d'Etude des Interactions Hôte Pathogène, Université d'Angers, Angers, France; 8 International Tuberculosis Research Center, Masan, Korea; 9 Institut Pasteur, Integrated Mycobacterial Pathogenomics, Paris, France; 10 Global Health Institute, Ecole Polytechnique Fédérale de Lausanne, Lausanne, Switzerland; Johns Hopkins School of Medicine, United States of America

## Abstract

A critical feature of *Mycobacterium tuberculosis*, the causative agent of human tuberculosis (TB), is its ability to survive and multiply within macrophages, making these host cells an ideal niche for persisting microbes. Killing the intracellular tubercle bacilli is a key requirement for efficient tuberculosis treatment, yet identifying potent inhibitors has been hampered by labor-intensive techniques and lack of validated targets. Here, we present the development of a phenotypic cell-based assay that uses automated confocal fluorescence microscopy for high throughput screening of chemicals that interfere with the replication of *M. tuberculosis* within macrophages. Screening a library of 57,000 small molecules led to the identification of 135 active compounds with potent intracellular anti-mycobacterial efficacy and no host cell toxicity. Among these, the dinitrobenzamide derivatives (DNB) showed high activity against *M. tuberculosis*, including extensively drug resistant (XDR) strains. More importantly, we demonstrate that incubation of *M. tuberculosis* with DNB inhibited the formation of both lipoarabinomannan and arabinogalactan, attributable to the inhibition of decaprenyl-phospho-arabinose synthesis catalyzed by the decaprenyl-phosphoribose 2′ epimerase DprE1/DprE2. Inhibition of this new target will likely contribute to new therapeutic solutions against emerging XDR-TB. Beyond validating the high throughput/content screening approach, our results open new avenues for finding the next generation of antimicrobials.

## Author Summary

Tuberculosis is still a major threat to global health. The disease in humans is caused by a bacterium, *Mycobacterium tuberculosis*, and treatment of an infected individual requires more than six months of chemotherapy. Because such a long course of treatment is required, compliance is low, which can result in the development of multidrug resistant strains (MDR-TB) and even extremely resistant strains (XDR-TB). Identifying new drug targets and potential lead therapeutic compounds are needed to combat MDR-XDR-TB. We developed a new type of assay based on the visualization of mycobacterium replication within host cells and applied it for the search of compounds that are able to chase the pathogen from its hideout. As a result, we found 20 new series of drug candidates that are effective against the bacilli in its hiding place, potentially addressing a crucial aspect in the resilience of the disease. We also showed that one series of compounds acts by inhibiting a key enzyme required for the synthesis of an essential component from the mycobacterial cell wall that is not targeted by any of the commercially available antituberculosis drugs. Altogether, our results pave the way for development of the next generation of antibacterial agents.

## Introduction

About one third of the world's population is estimated to be infected with *Mycobacterium tuberculosis*. In nine out of ten cases, *M. tuberculosis* persists in a latent state throughout an individual's lifetime [1]. The bacillus is found in a variety of host cells such as alveolar macrophages, dendritic cells and type II alveolar pneumocytes in infected lungs [2],[3],[4], as well as in adipocytes [5]. Whereas dendritic cells and adipocytes are not permissive for *in vitro* growth, *M. tuberculosis* replicates actively in macrophages and type II alveolar pneumocytes [2],[3],[5],[6]. The ability of *M. tuberculosis* to survive and multiply within host cells certainly contributes to the pathogenesis of tuberculosis (TB).

Though the exact means of ensuring intracellular survival is still a matter of debate [7],[8],[9], it is clear that potential new anti-tuberculosis drugs have to be active against *M. tuberculosis* inside host cells [10]. As this feature is not normally taken into account in traditional drug-screening procedures at an early stage, we developed a target-free cell-based assay suitable for high throughput screening that enables an unbiased search for compounds that kill intracellular *M. tuberculosis* without affecting the viability of the host macrophage. Such molecules would then serve as tools to identify novel druggable mycobacterial targets.

Target-based screens for antimicrobial agents have been disappointing to date [11],[12] whereas whole cell-based approaches with *M. tuberculosis* are fraught with logistic difficulties and hampered by long incubation periods. In this study, we developed a rapid phenotypic assay based on the use of automated confocal fluorescent microscopy to monitor intracellular growth of GFP-expressing *M. tuberculosis* H37Rv in Raw264.7 macrophages. The assay was set-up for the high throughput screening (HTS) of large chemical libraries in 384-well format and its robustness was validated with known antibiotics. By screening several thousand small molecules, new series of compounds were identified as well as some sharing structural similarities with known TB drugs. Among these, the benzamide series was then used as a bait to identify a new putative target. Using a combination of biochemical assays and genetic approaches, we showed that nitrobenzamide derivatives inhibited arabinan synthesis, which has not been observed for any of TB drugs so far. Altogether, these results demonstrate the feasibility of large scale screen for intracellular *M. tuberculosis* growth and open new avenues for enriching the TB drug pipeline as well as for finding new druggable targets.

## Results

### High Content Assay (HCA) Set-up based on the Monitoring of *M. tuberculosis* Infection in Macrophages

**Figure 1:**
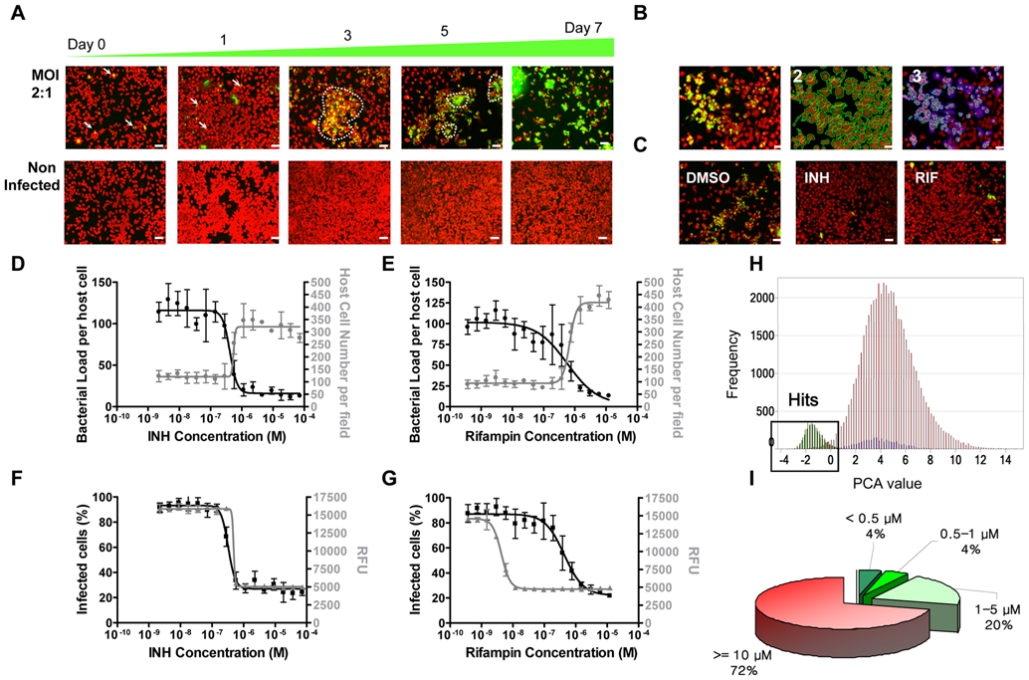
Monitoring of intracellular growth of tubercle bacilli inside macrophages by automated confocal microscopy and HT/CS screening results and hit profile. **(A)** Pictures of Raw264.7 cells infected with *M. tuberculosis* H37Rv-GFP at different time points after infection. NI: Non infected. Scale bar: 50 µm. **(B)** Infected Cell segmentation: 1: 2-color image; 2: cell mask detection, 3: purple cells correspond to infected cells. **(C)** Pictures of *M. tuberculosis* H37Rv-GFP infected Raw264.7 cells at day 5 in presence of INH, RIF at 1 µg/mL or DMSO control **(D–G)** INH and RIF pharmacology in intracellular (D, E) and *in vitro* (F ,G) assays. Bacterial load (arbitrary units, black circles), host cell number (gray circles) and percentage of infected cells (black squares) represent main parameters determined by our customized image analysis for the intracellular assay. *In vitro M. tuberculosis* growth is given in Relative Fluorescent Units (gray triangles). Results are representative of three independent experiments with standard deviation (SD) and have been reproduced more than 50 times during the screening. **(H)** Distribution of primary screening results after PCA-1x analysis. Yellow: INH 7 µM; Green: RIF 1.2 µM, blue: DMSO, red: screened compounds. **(I)** MIC range of the 486 confirmed hits.

To set up the optimal conditions of *M. tuberculosis* infection, Raw264.7 macrophages were first infected with mycobacteria that constitutively express green fluorescent protein (GFP) using different multiplicities of infection followed by kinetic analysis of intracellular bacterial growth. Confocal images of live samples were acquired using an automated confocal microscope (Opera™) over 7 days (**Figure 1A**). During the first twenty-four hours, a few discrete weakly fluorescent bacteria localized within the cells. At day 2, the average number of cells had increased and mycobacteria had started to spread into neighboring cells leading to zones of strongly fluorescent bacteria. At day 3, the number of cells had significantly diminished and the bacteria formed large, highly fluorescent aggregates, which covered almost the entire image from day 5 onwards. As a control, non-infected cells grew to confluence at day 2 and remained alive until day 5. Customized image analysis was developed to automatically quantify several different parameters such as the number of host macrophages, the percentage of infected cells and the average surface area of bacterial aggregates [13],[14]. Representative results of the cell segmentation method are displayed on **Figure 1B**. After two hours of infection, between 2 and 10% of Raw264.7 cells were found to harbor intracellular bacilli (**Figure S1A**). The percentage of infected cells steadily increased reaching 50% by day five with a MOI of 1. This augmentation correlated with substantial macrophage mortality due to the known cytopathogenic effects of *M. tuberculosis* [2] (**Figure S1B**). From day 5 to day 7, the percentage of infected cells continued to increase slowly up to 70%; however, the cell number dramatically decreased. Therefore to ensure that a sufficient number of cells was recorded in each field were recorded, the incubation time with *M. tuberculosis* was set at 5 days for the next series of experiments.

### Pharmacological Validation with Known Antitubercular Drugs

To validate the system, we first tested the effect of the standard anti-tuberculosis drugs such as isoniazid (INH) and rifampin (RIF) in our model (**Figure 1C**). As expected, these drugs demonstrated a dose-dependent decrease in both bacterial load and percentage of infected macrophages. Interestingly, an increase in host cell number was seen at effective concentrations, clearly demonstrating the ability of these drugs to prevent *M. tuberculosis* induced cytopathogenicity (**Figure 1D,E**). Taking into account both the amount of green fluorescent bacteria and the host cell number, this assay enables a dual and independent determination of intracellular anti-mycobacterial drug efficacy. In addition, the reference drugs were also applied against *M. tuberculosis* H37Rv-GFP grown in liquid broth without host cells. No major differences in minimal inhibitory concentrations (MIC) between the two assays were noticed for INH (**Figure 1F**), ethambutol, ethionamide and PA-824 [15] (**Table 1**), whereas RIF was 100-fold less efficient in the cell-based assay (**Figure 1G**), confirming the previously reported reduced activity of RIF against intracellular bacteria [16]. This finding clearly demonstrates that our dual read-out, cell-based drug screening system indeed measured the intracellular antibacterial activity of drugs, which allowed us to further adapt the system for High Throughput/Content Screening.

**Table 1:** Comparison of MICs of known TB drugs in broth and in intracellular growth conditions.

Compound Name	Mode of action/Target	MW	*In vitro* broth growth		Intracellular macrophage growth		
			MIC (µg/mL)	MIC (µM)	MIC (µg/mL)	MIC (µM)	Cell toxicity (µM)
Isoniazid	InhA, cell wall synthesis	137.1	0.16	1.2	0.16	1.2	>150
Ethionamide	Cell wall synthesis	166.2	1	6.0	1	6.0	120
Rifampin	RpoB, RNA polymerase	822.9	0.01	0.01	2.4	2.9	24
Rifabutin	RpoB, RNA polymerase	877.0	0.01	0.01	0.1	0.11	>20
Ethambutol	Cell wall synthesis	204.3	1	4.9	1	4.9	>20
PA-824	Hypoxia	359.3	0.08	0.22	0.08	0.22	>20
Pyrazinamide	cell wall synthesis	123.1	>20	>150	>20	>150	>150
Streptomycin	Protein synthesis	581.6	0.9	1.5	>20	>35	>35
Fusidic Acid	Protein synthesis	515.7	0.5	1	10	20	>20
Amikacin	Protein synthesis	585.6	0.04	0.07	>20	>150	>150
Levofloxacin	DNA gyrase	361.4	0.9	2.5	>10	>20	>20
AX20017	pknG	264.3	>5	>20	>5	>20	>20

### High Throughput/Content Screening (HT/CS)

A diverse library of 56,984 synthetic compounds was first screened at a single concentration. A normal distribution of the compounds was obtained using PCA-1x analysis (**Figure 1H**). 486 fully active hits were then confirmed by means of serial dilution experiments. The MIC of each hit was then determined using both the percentage of infected cells and the total cell number by taking advantage of the dual visual effect described above as an independent confirmation of compound activity. More than one-quarter of the hits (135 hits) had an MIC less than 5 µM, and 8% had a MIC below 1 µM, which is equivalent to that of INH (**Figure 1I**). A few compounds, such as compound CPD1, showed cytotoxicity at high concentrations as seen by a significant decrease in the cell number above 5 µM (**Figure S2C**).

Chemo-informatic cluster analysis of the 135 hits was performed and hits fell into 9 clusters plus 13 singletons (**Table S1**). The largest cluster had 69 members with an isonicotinohydrazide moiety similar to that of INH, used as a positive reference in our assay, which validated our approach. The second largest cluster of 24 derivatives shares a common benzamide scaffold. As no antimycobacterial effect had been previously reported for this particular chemical structure, a series of related derivatives was synthesized for further studies.

### Structure Activity Relationship (SAR) of Benzamides Derivatives

**Figure 2:**
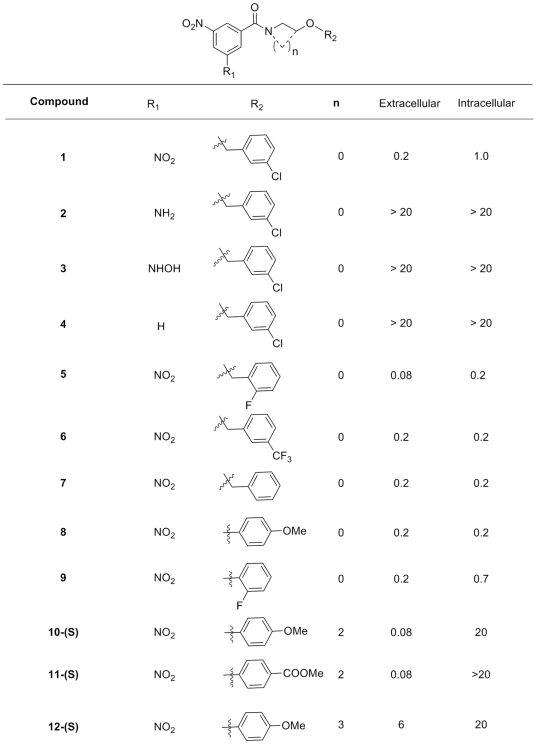
MIC (µM) for different nitrobenzamides against *M. tuberculosis* H37Rv growth. Extracellular and Intracellular correspond to growth in broth and in macrophages. DNB1 and DNB2 are compound 8 and 7 respectively.

To identify the chemical substituents necessary for benzamide antibacterial activity, over 155 additional derivatives were synthesized and their structure-activity relationship was analyzed using both our intracellular assay and the *in vitro* growth assay. The most potent compounds exhibited substitutions of the benzene moiety with a nitro group at positions 3 and 5 (**Figure 2** and **Figure S3**). The reduction of one nitro- to hydroxylamine and amino groups led to totally inactive compounds. In contrast, derivatives with an N-substitution by benzyloxy-ethyl or by phenoxy-ethyl showed enhanced activity with an MIC below 0.2 µM. More importantly, cyclic-benzamides had an MIC below 80 nM in the *in vitro* assay. However, these compounds turned out to be much less potent in the intracellular assay. Furthermore, substitution of the benzyloxy moiety by a chlorine- or fluorine atoms at position 3 led to increased potency in both assays in contrast to carboxyl substitutions. In parallel, we selected two compounds, *N*-(2-(4-methoxyphenoxy) ethyl)-3,5-dinitrobenzamide (DNB1) and ***N***-(2-(benzyloxy) ethyl)-3,5-dinitrobenzamide (DNB2), for further mechanistic studies and target identification (**Figure 2**).

### Dinitrobenzamides (DNB) Activity on Intracellular Growth of *M. tuberculosis* within Primary Macrophages and Cytotoxicity

**Figure 3:**
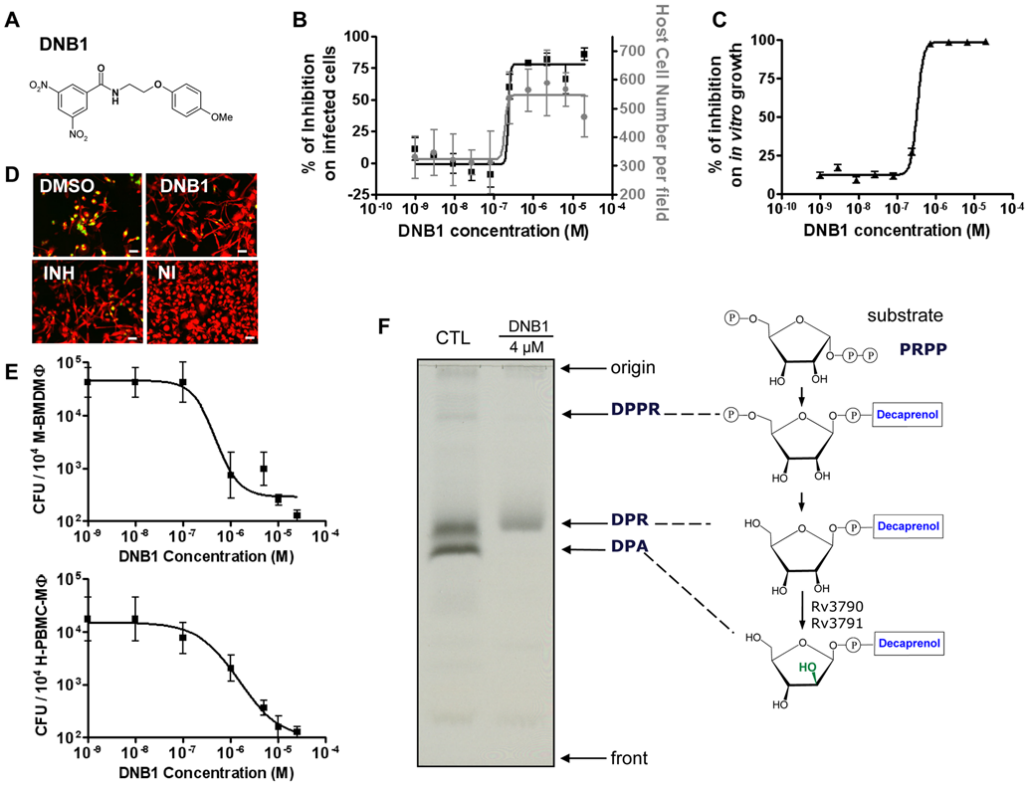
DNB1 activity confirmation and target identification. **(A)** Structure of DNB1 **(B)** Profile in intracellular assay from DNB1 of the benzamide scaffold; black squares and gray circles correspond to percentage of infected cells and host cell number **(C)** inhibition of the *in vitro* growth fluorescence assays by DNB1; black triangles correspond to relative fluorescent units. Results (mean+/−SD from 5 independent experiments) were normalized according to DMSO and INH control values. **(D)** Representative pictures of human primary macrophages infected with *M. tuberculosis* H37Rv-GFP (MOI 2.5∶1) at day 7 for DNB1 and INH (5 µM) and DMSO. NI: non infected. Scale bar corresponds to 50 µm. **(E)** Dose-response curve for DNB1 on *M tuberculosis* H37Rv-GFP infected mouse bone marrow-derived macrophages and human primary macrophages after 5 days of infection. Results are representative of 2 independent experiments. **(F)** Effect of DNB1 on the synthesis of decaprenyl-phospho-arabinose (DPA) by cell-free extracts of *M. smegmatis*. Bacterial extracts were treated with 4 µM (1.4 µg/mL) of DNB1 for 30 min at 37°C prior to the addition of p(^14^C)Rpp and ATP and further incubation for 90 min at 37°C. The same amount of reaction material was loaded as for the control (CTL). PRPP, 5-phosphoribosyl 1-pyrophosphate; DPPR, decaprenylphosphoryl 5-phosphoribose; DPR, decaprenyl-phospho-ribose.

DNB1 and DNB2 were pursued further since their activities on intra-cellular and extra-cellular *M. tuberculosis* were particularly favorable (**Figure 3A–C** and **Figure S3**). Their effects on primary macrophages were further determined. Host cells that had been pre-incubated with DNB1 harbored fewer bacteria compared to the DMSO control, and were more abundant at day 7 of infection as shown in **Figure 3D**. Conventional CFU determination was then performed after seven days of infection to quantify the remaining bacterial load. More than a ten-fold decrease in the number of CFUs was observed with both human and mouse primary cells at a DNB1 concentration above 5 µM (**Figure 3E**). Similar data were obtained for DNB2 (data not shown). This confirms the potency of this series of compounds. In parallel, no cell toxicity was noted for these compounds using conventional cytotoxicity assays of uninfected cells, indicating that our high content assay can reliably predict cytotoxicity (**Table S2**).

### Antimicrobial Effects of Dinitrobenzamides

Analysis of the broad antimicrobial spectrum was undertaken and revealed that the effect of these dinitrobenzamide derivatives was mainly restricted to actinomycetes with the most potent activity observed against *Mycobacterium* with an MIC of 75 ng/mL (0.2 µM) (**Table S2**). Of particular importance, DNB1 and DNB2 were also highly active against multidrug-resistant (MDR) and extensively drug-resistant (XDR) clinical isolates. Moreover, these two compounds were also associated with low levels of spontaneous resistance. Resistant mutants arose at frequencies between 1.2×10^−6^ and 1×10^−8^ on agar containing 2–16× the MICs of DNB1 or DNB2, a frequency similar to that with INH (**Table S3**). The potential for the development of resistance to dinitrobenzamides, *in vitro*, is therefore analogous to major anti-TB drugs.

Interestingly, the bactericidal effect on *M. tuberculosis* of DNB1 and DNB2 was found to be time-dependent (**Figure S4A**) and to require several days to reach bacterial clearance, implying that they could interfere with *de novo* mycobacterial component biosynthesis. This is further corroborated by the fact that the DNB compounds lost their activity in a non-replicating *M. tuberculosis* system [17]. Altogether these results suggested that the DNB compounds might act on different targets than current antituberculosis compounds.

### Effect of Dinitrobenzamides on Cell Wall Biosynthesis

To gain insight into the possible targets of dinitrobenzamides, we investigated the effect of DNB1 and DNB2 on the lipid composition of the cell envelope of *M. tuberculosis*; no effects on the biosynthesis of fatty acids, mycolic acids and/or other lipids were noted (data not shown). By contrast, DNB1 and DNB2 showed a clear-cut effect on the synthesis of the arabinan domains of arabinogalactan (AG) and lipoarabinomannan (LAM) (**Figure S4B,C**). Decaprenyl-phospho-arabinose (DPA) is the only known arabinofuranose (Ara*f*) donor in the biogenesis of AG and LAM in mycobacteria and is thus an essential precursor [18],[19]. To determine whether the effects of DNB were attributable to the inhibition of the synthesis of DPA or to that of DPA-dependent arabinosyltransferases involved in the elongation of both heteropolysaccharides, we set out to monitored DPA formation in treated and untreated extracts of *M. smegmatis* mc^2^155. Analyses revealed complete inhibition of DPA formation in the DNB-treated extracts concurrent with the accumulation of decaprenyl-phospho-ribose (DPR) (**Figure 3F**), indicating that the target of both DNB inhibitors is probably the heteromeric decaprenyl-phospho-ribose 2′ epimerase encoded by the *rv3790c (dprE1)/rv3791c* (*dprE2*) genes in *M. tuberculosis* H37Rv [20]. DprE1 has been recently described as the target of benzothiazinones (BTZ), a new class of antitubercular unrelated nitro-compounds [21]. BTZ- resistant mutants of *M. smegmatis* and *M. bovis* BCG were isolated and characterized as having a mutation in *dprE1*, in which Cysteine 387 had been replaced by a Glycine residue. This led us to test these *dprE1* mutants for their sensitivity to DNB1 and DNB2 (**Tables S4, S5**). They all displayed resistance to the DNB compounds corroborating our biochemical data (**Figure 3F**). These findings demonstrate the remarkable intracellular vulnerability of DprE1 and highlight the importance of pursuing the route of DPA production as a drug target.

## Discussion

Although the location and state of latent bacteria remains a matter of debate [22], one commonly shared hypothesis for mycobacterial persistence is that *M. tuberculosis* bacilli are able to survive in macrophages for prolonged periods of time and, unlike other bacteria, are able to actively replicate. It has clearly been established that the tubercle bacillus adopts a different phenotype in the host macrophage's phagosome compared to growth in extracellular conditions [7],[8]. The intraphagosomal transcription profile of *M. tuberculosis* is complex; a large variety of genes are over-expressed and temporally regulated in response to environmental cues. Altogether, this makes the identification of one specific factor in the tubercle bacillus that could be selected as the ideal drug target difficult. Consequently, non-target cell-based assays have emerged as a critical tool in the search for intracellular *M. tuberculosis* inhibitors.

Identification of antimycobacterial inhibitors active within host cells has long been limited due to cumbersome CFU plating, slow bacillary growth, safety requirements and difficulties in setting-up appropriate infection conditions. As a consequence, this approach was always used as a secondary assay after the initial selection of compounds that are active on broth grown bacteria. With the advent of automated confocal microscopy, the above-mentioned limitations could be circumvented and here we demonstrate the feasibility of large scale compound screening. To minimize the steps and to cope with HTS requirements, we performed suspension macrophage batch infection. To this end, careful attention was paid to remove the extracellular non-phagocytosed mycobacteria through the use of judicious centrifugation conditions and amikacin. Mycobacteria are able to grow independently of host cells and consequently any remaining extracellular bacilli would greatly compromise the validity of our model. Consequently, an extra amikacin treatment step was added to the protocol to further eliminate any remaining mycobacteria. Additional washing steps after amikacin treatment removed the antibiotic thereby minimizing the introduction of any bias towards compounds that could act synergistically with amikacin during screening. Thus with the optimized protocol, there are almost no non-phagocytosed mycobacteria left by the time compounds are added. Our results demonstrate that our assay specifically measured the effect of compounds on intracellular mycobacteria. Indeed, we observed weak inhibition with rifampin, an antibiotic that is known to be poorly active on intracellular mycobacteria. The reproducible 100-fold decrease in MIC for rifampin in the intracellular assay compared to the *in vitro* growth assay proved that the targeted bacteria are not extracellular. Otherwise no difference would have been seen in MIC between the two assays. As is well established and as we confirmed, macrophages are able to support high bacterial loads, which occupy a large part of the cell cytoplasm, eventually leading to macrophage cell death. Taking this into account, it was decided to set the data acquisition at day 5 post-infection when the cell number in the DMSO-control samples had significantly decreased relative to the antibiotic protected controls. Thus, monitoring cell number was an additional parameter enabling us to confirm the compound's antibacterial activity.

This confocal imaging-based assay could likely be adapted to other type of cells such as non-phagocytes in which *M. tuberculosis* is known to reside [3],[6]. Firstly, one could envision searching for drugs that will be active in different host settings. Secondly, our system could be adapted to the screening of compounds that target mycobacterial granulomas as these multi-cellular structures can be generated *in vitro* [23] and have been shown to promote infection [24].

One of the current challenges for TB drug discovery is the identification of compounds that are active against MDR and XDR bacteria. Compound-based approaches have lately proven to be effective for the development of new antitubercular drugs and have identified compounds with new mechanisms of actions such as TMC207 [25],[26]. The library of compounds that we screened contained more than 1,500 different heterocycles and was initially designed to be unbiased. This led to the identification of 23 clusters of molecules, among which the only known anti-tubercular compounds were INH derivatives. However, screening of another library led to a list of another set of hits including analogs of the nitroimidazopyran PA-824 [15] (data not shown). Also, our set of compounds does not include the typical chemical structures of some common antibacterials such as rifampin and streptomycin, which do not meet the Lipinski criteria on size and lipophilicity used for our library selection [27]. Taken together, this showed that the 57,000 member library does not contain the full repertoire of active small molecules.

The step-like shape of the dose-response curves (DRC) resulting from this cell-based phenotypic assay is unusual compared to the classical sigmoid profile of DRC for *in vitro* enzymatic or ligand-receptor type based assays. However, we can clearly rule out possible artifacts such as precipitation for several reasons. Firstly, our assay was calibrated with known TB inhibitors such as INH and ethambutol, which are water soluble and the curves obtained with these compounds displayed step-like shape. Secondly, the classical sigmoid with Hill coefficient value around 1 correspond to a fitting equation whose parameters are based on a model relying on the interaction of one unique substrate/ligand with one enzyme/receptor. In contrast, in our phenotypic assay, a large number of proteins are likely to be involved in the inhibition process, which may require compound intracellular uptake, pro-drug activation and target inhibition. In this system, the classical sigmoid model may not be the best fitting model. Thirdly, a similar step-like shape is observed for classical microbiological assays on whole mycobacterium such as for the rezasurin reduction assay. Thus, determination of MIC values as used for conventional TB drug susceptibility testing turned out to be more appropriate than half maximal inhibitory concentration IC50 measurement.

Structure-activity relationship studies were thus undertaken on the benzamide scaffold, which initially contained the largest number of molecules identified from the screen after the INH-like molecules. Cyclic-benzamides showed a 200-fold diminished intracellular growth inhibitory effect relative to its *in vitro* antibacterial effect, thereby demonstrating that compounds have to be efficiently taken up by cells to be effective against the intracellular bacillus. This intracellular assay may thus prove to be suitable for counter selection of compounds that have impaired membrane uptake.

Further development of the DNB series into lead compounds active *in vivo* requires improvement of their pharmacokinetics properties. Indeed, we observed that the nitro groups that are necessary for DNB antimycobacterial activity were very rapidly reduced into an amino group by mammalian liver enzymes. The mean half-life of the most active DNB compounds was about 8 minutes in a mouse microsomes assay and could already be significantly increased using encapsulation within nanoparticles. In a preliminary experiment using the acute mouse model of *M. tuberculosis*, a one log reduction of the CFU in the lungs of DNB treated animals compared to non-treated controls was observed after a three week daily treatment with 30 mg/kg/day following an intranasal infection (data not shown). Additional optimization of ADME properties and *in vivo* delivery of the DNB compounds is currently in progress.

Strikingly, chemical genomics identified decaprenyl phospho-ribose 2′-epimerase as the main target of dinitrobenzamides. This epimerase is encoded by *dprE1* and *dprE2* genes that are adjacent to the *embA-C* gene cluster whose products are also involved in the biosynthesis of LAM and AG and are targets of the first-line drug, ethambutol [28]. Consistent with their involvement in the synthesis of DPA, Rv3790c (DprE1) and Rv3791c (DprE2) have been suggested to be essential for the *in vitro* growth of *M. tuberculosis* as determined by transposon site hybridisation (TraSH) [29]. Interestingly, the fact that potent inhibitors of DprE1 could directly be isolated from the primary screening may indicate that this target is not only essential for bacterial growth inside the macrophages but also is easily accessible to small molecules. Though it is evident that the presence of the DNB scaffold in our library largely contributed to the identification of DrpE1 as a very druggable target, screening of another non-biased library could have resulted in similar findings. This is supported by the fact that as part of an independent study, DprE1 was recently identified as the target of benzothiazinones (BTZ), a different class of compounds that show potent antimycobacterial activity [21]. Moreover, BTZ-resistant mutants all displayed cross-resistance indicating that two chemically un-related nitro-compounds probably inhibit DPA production by the same mechanism. Further biochemical analyses will definitely contribute to a better understanding of the pharmacology of this new druggable mycobacterial target.

The mechanisms of action of the other scaffolds found in this study remains to be characterized and will likely contribute to the discovery of new bacterial as well as cellular targets. For example, derivatives from Scaffold IX (**Table S1**) are effective against XDR isolates and have no effect against DprE1 activity and ATP synthesis, which suggests that they may act on an unknown target. In addition, molecules sharing Scaffold III displayed selective inhibition of intracellular growth within macrophages, raising the possibility that a host cellular target could be involved in the antibacterial effect. Alternatively, using other libraries could lead to the identification of scaffolds with different chemical structures. For instance, screening another set of 120,000 molecules in our cell-based assay revealed analogs of the nitroimidazopyran PA-824 [15], which was shown to induce bacterial killing by nitric oxide release [30]. Taken together this clearly shows that both the repertoire of druggable targets and potential antitubercular compounds has not yet been fully uncovered. Finally, we would like to point out that high throughput/content screening is a powerful generic approach that can be used to discover inhibitors for other intracellular pathogens that are genetically tractable.

## Methods

### Chemical Compounds

The 56,984-compound library was purchased from Timtec (26,500 molecules), Cerep (10,484) and ChemBridge™ (20,000) and each sub-library consisted of a selection of molecules based on their chemical diversity and drug-like properties. An in-house evaluation showed that ≥80% of the compounds met the criteria of the ‘rule of 5’ of Lipinski [31]. Small molecules from the screening libraries, CPD1, *N*-(2-(4-methoxyphenoxy) ethyl)-3,5-dinitrobenzamide (DNB1) and ***N***-(2-(benzyloxy) ethyl)-3,5-dinitrobenzamide (DNB2) were dissolved in pure DMSO (Sigma, D5879) and added to the assay plates using an EVObird liquid handler (PerkinElmer) to reach a final concentration of 20 µM.

### Mycobacterial Strains and *In Vitro* Bacterial Growth Assay

The description of all the mycobacterial strains used in this study is given in **Table S6**. *Mycobacterium tuberculosis* H37Rv, H37Ra and BCG Pasteur were used as reference strains. The recombinant strain of *M. tuberculosis* H37Rv expressing the green fluorescent protein (H37Rv-GFP) bears an integrative plasmid (based on Ms6) carrying a *gfp* gene constitutively expressed from the promoter *pBlaF* [32]. All strains were precultured at 37°C in Middlebrook 7H9 broth (Difco) supplemented with 0.05% Tween 80 (Sigma, P8074) and oleic acid-albumin-dextrose-catalase (OADC) for 14 days. 384-well plates (Greiner, #781091) were first preplated with 0.5 µl of compound dispensed by EVOBird (Evotec) in 10 µl of Middlebrook 7H9-OADC medium supplemented with 0.05% Tween 80. Forty microliters of H37Rv-GFP bacterial suspension diluted to 2×10^6^ CFU/mL (based on GFP fluorescence assessment and a reference curve) was then added to the diluted compound resulting in a final volume of 50 µl containing 1% DMSO. Plates were incubated at 37°C, 5% CO_2_ for 7 days. Mycobacterial growth was determined by measuring GFP-fluorescence using a Victor 3 reader (Perkin-Elmer Life Sciences). The resazurin reduction method was used for reference strains, MDR, XDR and clinical isolates [33]. Isoniazid at 0.05 µg/mL and 1 µg/mL (Sigma, I3377), Rifampin at 1 µg/mL (Euromedex) and DMSO were used as controls. Drug susceptibility testing on benzothiazinone-resistant mycobacteria with various mutations in the *rv3790* gene was performed as recently reported [21].

### Macrophage Infection Assay in 384-Well Plates, Image Acquisition and Analysis

384-well Evotec plates (#781058) were first preplated with 0.5 µl of compound dispensed by EVOBird (Evotec) in 10 µl of RPMI 1640 (Gibco) supplemented with 10% heat-inactivated fetal calf serum (FCS, Gibco). Raw 264.7 (ATCC # TIB-71) (1.5×10^8^ cells) were infected with H37Rv-GFP [32] in suspension at a MOI of 1∶1 in RPMI 1640 supplemented with 10% heat-inactivated FCS for 2 hours at 37°C with shaking. After two washes by centrifugation, the remaining extracellular bacilli in the infected cell suspension were killed by a 1 hour Amikacin (20 µM, Sigma, A2324) treatment. After a final two-wash centrifugation, 10 000 infected cells were dispensed into each plate well pre-plated with compounds and controls. Infected cells were then incubated for 5 days at 37°C, 5% CO_2_. After five days, macrophages were stained with SYTO 60, 5 µM (Invitrogen, S11342) for 1 hour at 37°C and image acquisition was performed on an EVOscreen-MarkIII fully automated platform (PerkinElmer) integrated with an Opera™ (20X-water objective, NA 0.70) and located in a BSL-3 safety laboratory. Mycobacteria-GFP were detected using a 488-nm laser coupled with a 535/50 nm detection filter and SYTO 60 labelled cells with a 635-nm laser coupled with a 690/40 nm detection filter. Four fields were recorded for each plate well and each image was then processed using dedicated in-house image analysis software (IM) described elsewhere [14]. Briefly, the algorithm first segments the cells on the red channel using a sequence of processing steps [13]. Firstly the contour of each macrophage is delineated using an algorithm based on the intensity signal given by the red channel (**Figure 1B**). The number of red delineated surfaces corresponds to the number of macrophages. The host cell is then considered to be infected by *M. tuberculosis* if there is an overlap of at least 3 pixels in the green channel above a given intensity threshold within the cell surface. The ratio of infected cells to the total number of cells determines the percentage of infected cells. Another parameter deduced from the images is the bacterial load that refers to the total surface area of all the green objects that partly cover the delineated macrophages.

### Primary Component Analysis (PCA) for High Content Analysis

Eight parameters that include cell number, cell surface, infected cell number, number of green objects, green object intensity, green object surface, green surface in infected cells and infection ratio were then processed plate by plate in a PCA protocol developed using PipelinePilot™ (Accelrys). Briefly, the values from both positive and negative controls of each plate were first used to create a PCA model in 1 dimension for the plate (Minimum Variance explained = 0.75, Center-and-Scale data pre-transformation). The model was then applied to calculate the new coordinates of compounds and controls for that plate. Similar analysis was then repeated for each new screened plate. A Z′ score was then calculated, and controls and plates were accepted with a Z′ score above 0. For each plate, the model described more than 99% of the variance. PCA-1x analysis improved active compound separation compared to the analysis based on the infected cell percentage parameter as demonstrated by achievement of better Z′ values (**Figure S2B**). Hits were selected with a PCA-1x value below 0.5, corresponding to the separation value between the DMSO and the INH 1 µg/mL populations.

### Primary Screen and Hit Confirmation

The compound library was screened at a single concentration of 20 µM. Hits were then cherry-picked and tested in ten- 2-fold serial dilutions (from 20 µM to 0.5 nM) in duplicate. 486 hits (0.85%) were then confirmed.

### Statistics

Data obtained from either the intracellular assay image analysis or from the conventional antibacterial assay were then processed using ActivityBase (IDBS) to calculate statistical data (% of inhibition, Z score for each compound, Z′, coefficient of variation (CV) etc. for the control plates). Results visualization was performed with Spotfire (Tibco). If not specified in the figure legend, data are expressed as mean+/−SD from 2 independent experiments.

### Infection of Primary Macrophages and CFU Determination

Mouse bone-marrow-derived macrophages were obtained by seeding 10^7^ bone marrow cells from C57BL/6 mice in 75 cm^2^ dishes in RPMI 1640 (Gibco™) supplemented with 10% heat-inactivated FCS and 10% L-cell conditioned medium (L-929). Peripheral Blood Mononuclear Cells (PBMC) were isolated from buffy coat from healthy volunteers. 15 ml of Ficoll-Paque Plus (Amersham Biosciences, Sweden) were added to PBS diluted buffy coat diluted and centrifuged at 2500×*g* for 20 min. PBMC were obtained by CD14^+^ beads separation (Miltenyi Biotec, Germany), washed 3-times with PBS containing 1% FCS and transferred to 75 cm^2^ culture flask containing RPMI 1640 media, 10% FCS and 50 ng/ml of recombinant-human macrophage colony stimulating factor (rh-MCSF, R & D systems, Minneapolis). After 6 days, murine or human macrophages were harvested with Versene (Gibco™) and seeded at a density of 1.5×10^4^ cells per well in 384-well Evotec plates in 50 µl RPMI 1640 supplemented with 10% heat-inactivated FCS and 10% L-929 or 50 ng/ml of rh-MCSF respectively. Adherent cells were then infected with bacterial suspensions at a MOI of 2.5 to 1 bacteria per cell and incubated for 2 h. Cells were then washed three times with PBS supplemented with 1% FCS and further incubated with different concentration of DNB compounds for 7 days. Cells were then lysed with 0.1% Triton X-100 (Sigma) in H_2_O and serial dilutions were performed to quantify CFUs as previously reported [34].

### Frequency of Spontaneous Resistance

The frequency of spontaneous mutation was determined on 7H10-OADC plates containing increasing concentrations of DNB1 and DNB2 at 0.4, 0.8, 1.6 and 3.2 µM. 10^5^, 10^6^, 10^7^ and 10^8^ CFU containing bacterial suspensions were spread on dinitrobenzamides containing agar plates. After 5–6 weeks at 37°C, colonies were counted and frequency of mutation was evaluated as the ratio of colonies grown relative to the original inoculum. DMSO and INH were used as negative and positive controls respectively.

### Effects of Inhibitors on the Formation of DPA *In Vitro*

Reaction mixtures contained 1 mg of *M. smegmatis* membrane and cell wall (P60) proteins [20], 80 µM ATP, 120,000 dpm p(^14^C)Rpp [35], 50 mM MOPS pH 7.9, 5 mM 2-mercaptoethanol and 10 mM MgCl_2_. Reactions were stopped by the addition of CHCl_3_∶CH_3_OH (2∶1) and the organic phase backwashed with CHCl_3_∶CH_3_OH∶H_2_O (3∶47∶48). After drying under N_2_, the radiolabeled material was dissolved in CHCl_3_∶CH_3_OH∶H_2_O∶NH_4_OH (65∶25∶3.6∶0.5) for TLC analysis.

### Effects of Inhibitors on Mycobacterial Cell Wall Synthesis

For assessing the effects of DNB1 and DNB2 on whole *M. tuberculosis* H37Ra, 0.6 to 80 µM (0.2 to 28 µg/mL respectively) of the compounds were added to bacterial cultures grown to mid-log phase in glycerol-alanine-salts medium and incubated for 16 hrs at 37°C with shaking, after which 1 µCi/mL [U-^14^C]glucose (specific activity, 317 Ci mol^−1^, MP Biomedicals Inc.) or 0.5 µCi/mL [1,2-^14^C]acetic acid (specific activity, 60 Ci mol^−1^, NEN Radiochemicals) were added and the cultures were incubated for another 24 hrs at 37°C. Untreated and inhibitor-treated bacteria were collected by centrifugation, washed and their lipids, lipoglycans (LM and LAM) and mycolyl-arabinogalactan-peptidoglycan (mAGP) complex, were extracted essentially as described [36]. ^14^C-glucose- and ^14^C-acetate-derived lipids and fatty acids (including mycolates) were analyzed by TLC on silica gel 60 aluminum-backed plates (Merck, Darmstadt, Germany) in a variety of solvent systems [37]. ^14^C-glucose-derived lipoglycans were separated on Tricine gels, transferred to nitrocellulose membranes and revealed by autoradiography. The amount of radioactivity incorporated into the individual sugars of the mAGP complex was determined by hydrolysis of the ^14^C-glucose-derived material with 2M CF_3_COOH for 3 hrs at 120°C and separation of the individual monosaccharides (upon removal of fatty acids) on aluminum-backed TLC plates developed twice in pyridine∶ethyl acetate∶acetic acid∶water (5∶5∶1∶3). Autoradiograms were produced by exposure of the TLCs and nitrocellulose membranes to KODAK-Biomax MR films at −70°C.

## Supporting Information

Figure S1 Quantification of M. tuberculosis growth into macrophages by automated confocal imaging. Image-based quantification of (A) percentage of infected cells and (B) the total number of cells from 2 hours to day 7 after infection with H37Rv-GFP at a multiplicity of infection of 0.5 (gray squares), 1 (black circles) and 2 (dark gray triangles). Non-infected cells (black diamonds) were used as the negative control. Results are representative of 2 independent experiments.
(0.04 MB PDF)

Figure S2 Overview of large scale screening on the M. tuberculosis infected macrophages. (A) Large-scale batch-based macrophage infection assay sequence. (B) Comparison of Z′ score (DMSO vs INH 7 µM (1 µg/mL)) calculated with percentage of Infected Cells (x-axis, classical method) or after PCA-1x analysis (y-axis). The line represents the y = x equation. Most of the Z′ scores calculated after PCA-1x analysis are higher demonstrating a better separation between active and non-actives compounds. (C) Hit CPD1 profile in the intracellular assay showing major cytotoxicity above 5 µM. Results (mean+/−SD from 2 independent experiments) were normalized according to DMSO and INH control values. Black squares and gray circles correspond to the percentage of infected cells and the host cell number respectively as determined by our customized image analysis for the intracellular assay.
(0.16 MB PDF)

Figure S3 Dose-response analysis of Compounds 1 to 12-(S) listed in Table 2 in *in vitro* intracellular and in broth grown bacterial assays. Percentage of inhibition of intracellular growth from infected cells parameter (black squares) and extracellular growth (gray triangles) Results are shown as the mean of 2 independent experiments with standard deviation (SD).
(0.09 MB PDF)

Figure S4 DNB1 and DNB2 exhibited a time dependent inhibitory effect and inhibited M. tuberculosis arabinans biosynthesis. (A) Kinetics of DNB1 (3 µM, black triangles and DNB2 at (3 µM, black circles) bactericidal activity on *M. tuberculosis* H37Rv growth *in vitro*. DMSO-treatment was used a control (gray squares). Effect of DNB1 and DNB2 on the synthesis of the arabinan domains of arabinogalactan (B) and LAM (C) in *M. tuberculosis*. After incubation of bacterial cultures with 40 and 80 µM (14 µg/mL and 28 µg/mL) of compounds as described in Materials and Methods, lipoglycans and cell wall monosaccharides were purified before being loaded onto TLC. Equal volumes and cpm counts were loaded for lipoglycans and cell wall monosaccharides respectively, for control (CTL) and samples. Monosaccharides were identified by co-migration with commercial standards (Ara, arabinose; Gal, galactose; Glc, glucose). Results are representative of 2 independent experiments.
(0.15 MB PDF)

Table S1 Chemo-informatic cluster analysis of the 135 confirmed hits
(0.04 MB PDF)

Table S2 Cytotoxicity and antibacterial spectrum of DNB1 and DNB2
(0.02 MB PDF)

Table S3 Proportion of spontaneous resistant mutants for DNB1 and DNB2
(0.02 MB PDF)

Table S4 DNB effect on *M. smegmatis* mc^2^ 155 mutants in DprE1
(0.01 MB PDF)

Table S5 DNB effect on *M. bovis* BCG mutants in DprE1
(0.01 MB PDF)

Table S6 List of mycobacterial strains used in this study
(0.01 MB PDF)
