# Supplementary figures and images for: High Content Screening Identifies Decaprenyl-Phosphoribose 2′ Epimerase as a Target for Intracellular Antimycobacterial Inhibitors
Source: PLoS Pathog. 2009 Oct 30;5(10):e1000645. doi: 10.1371/journal.ppat.1000645 (PMC2763345; doi:10.1371/journal.ppat.1000645)

**A**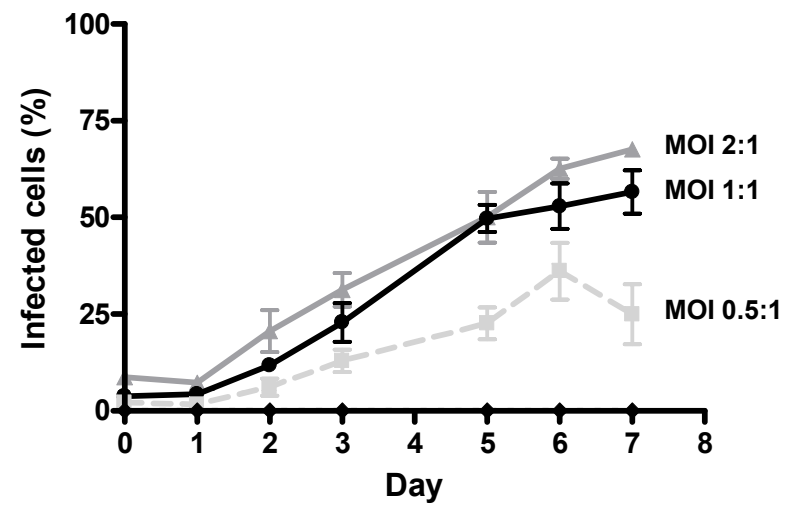**B**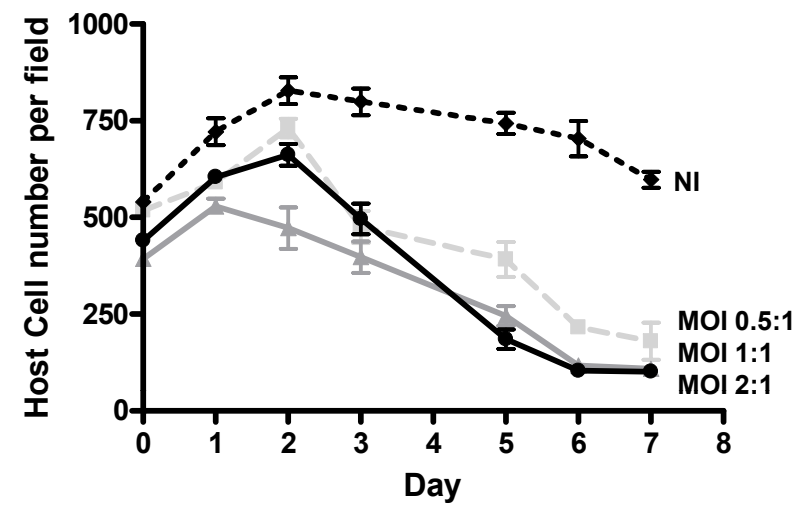

Supplement: Figure S1 — Quantification of M. tuberculosis growth into macrophages by automated confocal imaging. Image-based quantification of (A) percentage of infected cells and (B) the total number of cells from 2 hours to day 7 after infection with H37Rv-GFP at a multiplicity of infection of 0.5 (gray squares), 1 (black circles) and 2 (dark gray triangles). Non-infected cells (black diamonds) were used as the negative control. Results are representative of 2 independent experiments. (0.04 MB PDF) [file ppat.1000645.s001.pdf]

**A**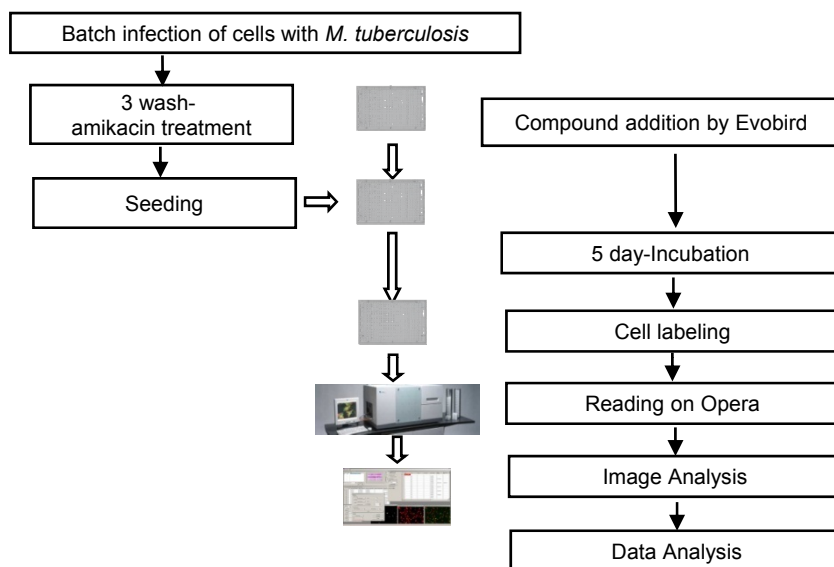**B**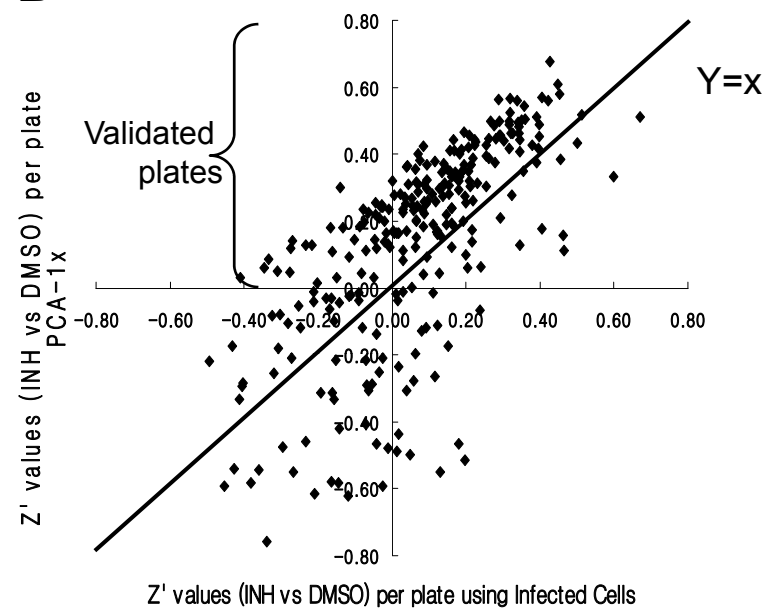**C**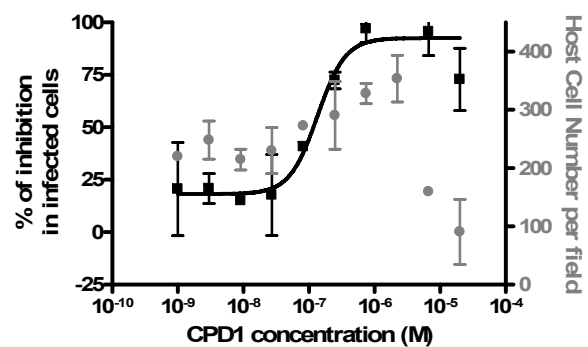

Supplement: Figure S2 — Overview of large scale screening on the M. tuberculosis infected macrophages. (A) Large-scale batch-based macrophage infection assay sequence. (B) Comparison of Z′ score (DMSO vs INH 7 µM (1 µg/mL)) calculated with percentage of Infected Cells (x-axis, classical method) or after PCA-1x analysis (y-axis). The line represents the y = x equation. Most of the Z′ scores calculated after PCA-1x analysis are higher demonstrating a better separation between active and non-actives compounds. (C) Hit CPD1 profile in the intracellular assay showing major cytotoxicity above 5 µM. Results (mean+/−SD from 2 independent experiments) were normalized according to DMSO and INH control values. Black squares and gray circles correspond to the percentage of infected cells and the host cell number respectively as determined by our customized image analysis for the intracellular assay. (0.16 MB PDF) [file ppat.1000645.s002.pdf]

**A**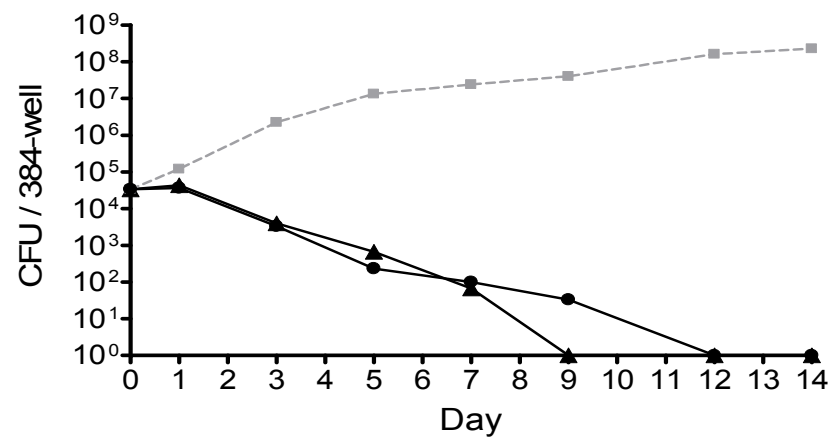**B**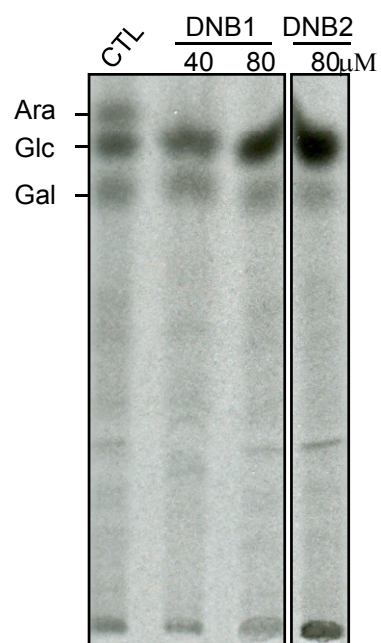**C**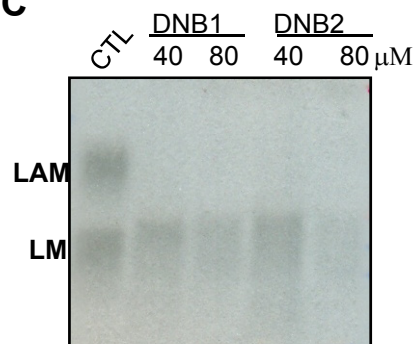

Supplement: Figure S4 — DNB1 and DNB2 exhibited a time dependent inhibitory effect and inhibited M. tuberculosis arabinans biosynthesis. (A) Kinetics of DNB1 (3 µM, black triangles and DNB2 at (3 µM, black circles) bactericidal activity on M. tuberculosis H37Rv growth in vitro. DMSO-treatment was used a control (gray squares). Effect of DNB1 and DNB2 on the synthesis of the arabinan domains of arabinogalactan (B) and LAM (C) in M. tuberculosis. After incubation of bacterial cultures with 40 and 80 µM (14 µg/mL and 28 µg/mL) of compounds as described in Materials and Methods, lipoglycans and cell wall monosaccharides were purified before being loaded onto TLC. Equal volumes and cpm counts were loaded for lipoglycans and cell wall monosaccharides respectively, for control (CTL) and samples. Monosaccharides were identified by co-migration with commercial standards (Ara, arabinose; Gal, galactose; Glc, glucose). Results are representative of 2 independent experiments. (0.15 MB PDF) [file ppat.1000645.s004.pdf]
