# Supplementary material for: High Content Screening Identifies Decaprenyl-Phosphoribose 2′ Epimerase as a Target for Intracellular Antimycobacterial Inhibitors
Source: PLoS Pathog. 2009 Oct 30;5(10):e1000645. doi: 10.1371/journal.ppat.1000645 (PMC2763345; doi:10.1371/journal.ppat.1000645)
Supplement: Table S1 — Chemo-informatic cluster analysis of the 135 confirmed hits (0.04 MB PDF) [file ppat.1000645.s005.pdf]

Table S1 Chemo-informatic cluster analysis of the 135 confirmed hits

Scaffold Name	Scaffold Coding	Number of Compounds	Most Representative Molecule
Isonicotinohydrazides	I	69	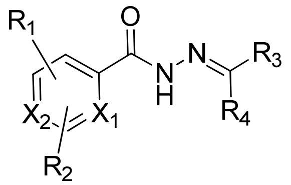
Benzamides	II	24	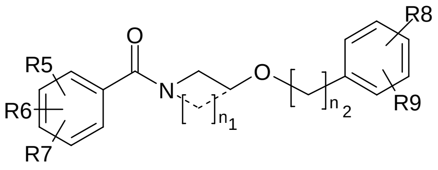
Thiazolhydrazones	III	6	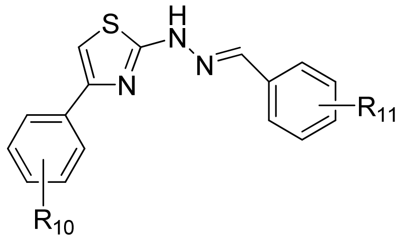
Hydrazinecarbothioamides	IV	5	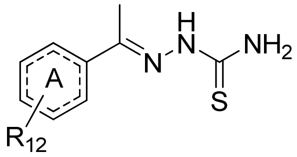
Phenylurea	V	5	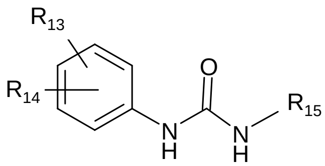
Cinnamides	VI	4	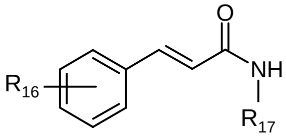
Furancarbohydrazides	VII	4	

			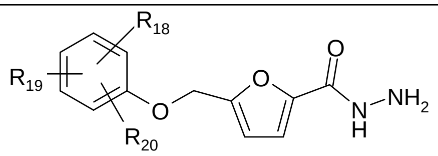
Thiophenes	VIII	3	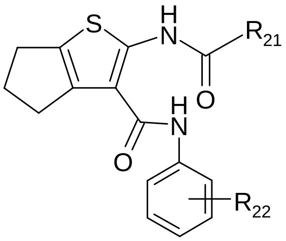
Pyrazole-pyridines	IX	2	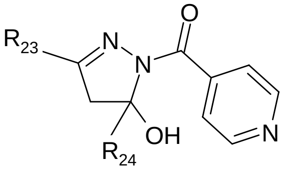
Pyridopyrimidinone	X	1	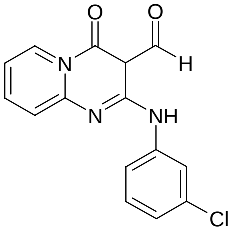
Singleton	XI	1	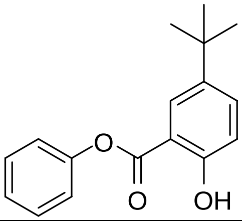
Singleton	XII	1	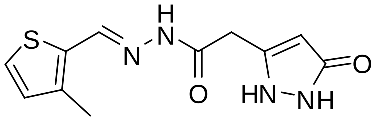

Singleton	XIII	1	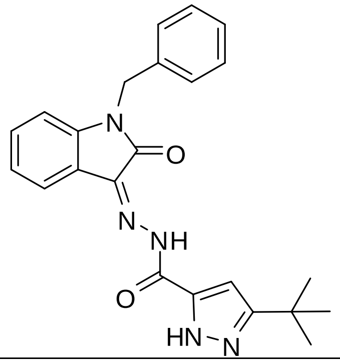
Singleton	XIV	1	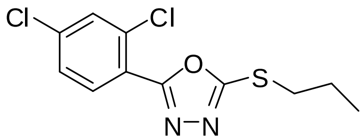
Singleton	XV	1	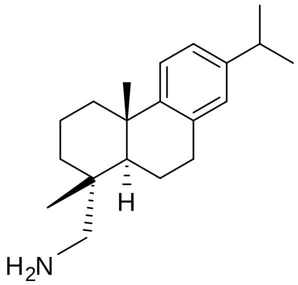
Singleton	XVI	1	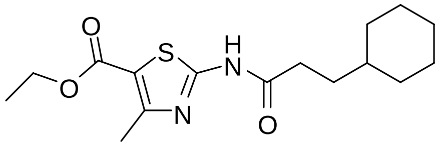
Singleton	XVII	1	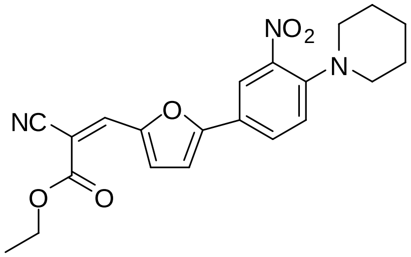
Singleton	XVIII	1	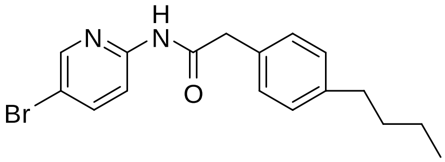

Singleton	XIX	1	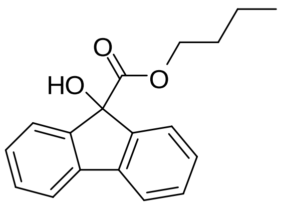
Singleton	XX	1	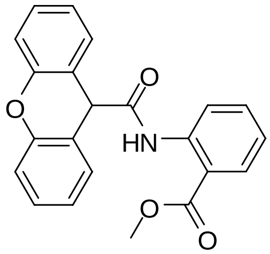
Singleton	XXI	1	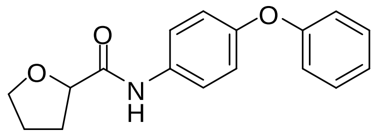
Singleton	XXII	1	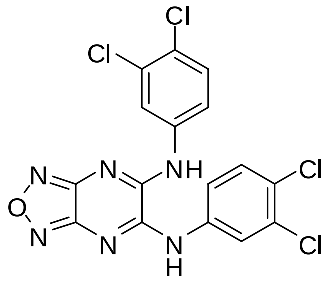

X_1=H, X_2=N, R_x =H unless specified; R_4=2-fluorophenyl; R_5=2-nitro ; R_7=4-nitro; R_8=4-methoxy; n_1= 0; n_2=1 ; R_{11}=4-methoxy; A=phenyl, R_{12}=4-isobutyl; R_{13}=3-Cl ; $R_{14=}$2-CF_3; $R_{15,}$ = 7,7 dimethylbicycloheptyl ; R_{16}=dioxolane ; R_{17}= 7,7 dimethylbicycloheptyl ; R_{18}=4-Cl ; R_{21}=2-chlorophenyl ; R_{22}=2-chlorophenyl ; R_{23}=ethyl ; R_{24}=CF_3.
