# Supplementary material for: High Content Screening Identifies Decaprenyl-Phosphoribose 2′ Epimerase as a Target for Intracellular Antimycobacterial Inhibitors
Source: PLoS Pathog. 2009 Oct 30;5(10):e1000645. doi: 10.1371/journal.ppat.1000645 (PMC2763345; doi:10.1371/journal.ppat.1000645)
Supplement: Table S2 — Cytotoxicity and antibacterial spectrum of DNB1 and DNB2 (0.02 MB PDF) [file ppat.1000645.s006.pdf]

Table S2 Cytotoxicity and Antibacterial spectrum of DNB1 and DNB2

**Cytotoxicity**				
**Compounds**			DNB1	DNB2
**Host Cells**			Range of IC50 (μM)	
SK-N-SH -Brain			>100	>100
HepG2-Hepatocytes			>100	>100
MRC5- Lung			>100	>100
BJ- Skin			>100	>100
HEK293- Kidney			>100	>100
Jurkat -T-cell			50	50
THP-1 - Monocytes			50	50
Primary BMDM			50	50
Primary human macrophages			50	50
**Antibacterial activity & Specificity**				
Mycobacterium				
Strains/Isolates	Type	Number	Range of MICs for multiple strains (μM)	
M. tuberculosis clinical isolates[1]	Drug Sensitive	1	0.38	0.31
	RIF^R	4	0.02-0.05	0.08
	$Kana^R$	1	0.05-0.1	0.04-0.08
	XDR	5	0.02-0.05	0.04-0.08
	MDR	8	0.05-0.1	0.04-0.08
M. tuberculosis laboratory strains	H37Rv		0.2	0.2
	H37Ra		0.2	0.2
	BCG Pasteur-Tokyo		0.2	0.2-0.7
M. smegmatis	mc^2 155		0.4	0.4
Gram-negative				
Acinetobacter baumannii, Escherichia coli, Enterobacter cloacae, E. aerogenes, Klebsiella oxytoca, Pseudomonas aeruginosa, Salmonella enteridis, Vibrio mimicus			>250	>250
Gram-positive				
Staphylococcus aureus, S. epidermis, S. capitis, S. xylosus, Micrococcus luteus, Listeria innocua, Lactobacillus gallinarum, group G Streptococcus, Streptococcus agalactiae, S. pyogenes, Enterococcus faecalis, E. faecium, E. gallinarum, Bacillus pumilus			>250	>250
Corynebacterium				
C. striatum			27	27
C. jeikeium			2.7	2.7
Fungi				
Candida albicans, C. glabrata, C. parapsilosis			>250	>250

RIF: Rifampin, Kana: Kanamycin, ^{R}: resistant. [1]The clinical isolates were isolated either from resected lung tissue or sputum specimen, which were collected from active tuberculosis in-patients from the National Masan Tuberculosis Hospital during October 2003 to March 2007
