# Supplementary material for: High Content Screening Identifies Decaprenyl-Phosphoribose 2′ Epimerase as a Target for Intracellular Antimycobacterial Inhibitors
Source: PLoS Pathog. 2009 Oct 30;5(10):e1000645. doi: 10.1371/journal.ppat.1000645 (PMC2763345; doi:10.1371/journal.ppat.1000645)
Supplement: Table S3 — Proportion of spontaneous resistant mutants for DNB1 and DNB2 (0.02 MB PDF) [file ppat.1000645.s007.pdf]

Table S3 Proportion of spontaneous resistant mutants for DNB1 and DNB2

Compound	Concentration of compound (fold-MIC)	Number of revertants per plate inoculated with *M. tuberculosis* (CFU)				Frequency of spontaneous resistance
		10^5	10^6	10^7	10^8	
DNB1	2X	-	-	12	123	$1.2x10^{-6}$
	4X	-	-	1	80	$8x10-^{7}$
	8X	-	-	-	32	$3.2x10-^{7}$
	16X	-	-	-	1	$1x10^{-8}$
DNB2	2X	-	-	7	136	$1.3x10^{-6}$
	4X	-	-	-	72	$7.2x10^{-7}$
	8X	-	-	-	26	$2.6x10^{-7}$
	16X	-	-	-	1	1×10^{-8}
INH-control	16X	ND	ND	33	ND	3×10^{-6}

ND: not done; -: no colonies
