# Supplementary material for: High Content Screening Identifies Decaprenyl-Phosphoribose 2′ Epimerase as a Target for Intracellular Antimycobacterial Inhibitors
Source: PLoS Pathog. 2009 Oct 30;5(10):e1000645. doi: 10.1371/journal.ppat.1000645 (PMC2763345; doi:10.1371/journal.ppat.1000645)
Supplement: Table S4 — DNB effect on M. smegmatis mc2 155 mutants in DprE1 (0.01 MB PDF) [file ppat.1000645.s008.pdf]

Table S4 DNB effect on *M. smegmatis* mc^2 155 mutants in DprE1

MIC (µg/mL)	DNB1	DNB2	RIF
M. smegmatis mc^2 155 Wild Type	0.15	0.25	0.31
Rv3790/DprE1 Cys387Gly (MN47)	100	50	0.31
Rv3790/DprE1 Cys387Ser (MN84)	>100	>100	0.31
