# Supplementary material for: High Content Screening Identifies Decaprenyl-Phosphoribose 2′ Epimerase as a Target for Intracellular Antimycobacterial Inhibitors
Source: PLoS Pathog. 2009 Oct 30;5(10):e1000645. doi: 10.1371/journal.ppat.1000645 (PMC2763345; doi:10.1371/journal.ppat.1000645)
Supplement: Table S5 — DNB effect on M. bovis BCG mutants in DprE1 (0.01 MB PDF) [file ppat.1000645.s009.pdf]

Table S5 DNB effect on *M. bovis* BCG mutants in DprE1

MIC (µg/mL)	DNB1	DNB2	RIF
BCG<br>Wild Type	0.075	0.25	0.31
Rv3790/DprE1<br>Cys387Ser<br>(BN2)	>10	>10	0.31
