# Supplementary material for: High Content Screening Identifies Decaprenyl-Phosphoribose 2′ Epimerase as a Target for Intracellular Antimycobacterial Inhibitors
Source: PLoS Pathog. 2009 Oct 30;5(10):e1000645. doi: 10.1371/journal.ppat.1000645 (PMC2763345; doi:10.1371/journal.ppat.1000645)
Supplement: Table S6 — List of mycobacterial strains used in this study (0.01 MB PDF) [file ppat.1000645.s010.pdf]

Table S6 List of mycobacterial strains used in this study

Mycobacterial type strain (origin)	Antibiotic Resistance Pattern	Purpose in this study
M. tuberculosis H37Rv (Lab.Institut Pasteur http://genolist.pasteur.fr/TubercuList/)	S	DST
M. tuberculosis H37Rv expressing eGFP (Lab)	Hygromycin	Screening
M. tuberculosis H37Ra (Lab)	S	DST
M. tuberculosis 20031014 (Tissue)	MDR+A	DST
M. tuberculosis 20040511 (Tissue)	MDR+Q	DST
M. tuberculosis 20041005 (Tissue)	MDR	DST
M. tuberculosis 20041116 (Tissue)	MDR+A	DST
M. tuberculosis 20050104 (Tissue)	MDR+A	DST
M. tuberculosis 20060523 (Tissue)	Rif	DST
M. tuberculosis 20060620 (Tissue)	A	DST
M. tuberculosis 20061024 (Tissue)	S	DST
M. tuberculosis 20070327 (Tissue)	Rif	DST
M. tuberculosis DR06057 (Sputum)	XDR	DST
M. tuberculosis MDR03003 (Sputum)	MDR	DST
M. tuberculosis MDR03009 (Sputum)	XDR	DST
M. tuberculosis MDR04011 (Sputum)	MDR+A	DST
M. tuberculosis MDR04032 (Sputum)	XDR	DST
M. tuberculosis MDR04073 (Sputum)	XDR	DST
M. tuberculosis MDR05018 (Sputum)	MDR	DST
M. tuberculosis MDR05020 (Sputum)	Rif	DST
M. tuberculosis MDR05038 (Sputum)	XDR	DST
M. tuberculosis MDR05043 (Sputum)	Rif	DST
M. bovis BCG Pasteur (Vaccine)	S	DST
M. bovis BCG Pasteur, BN2 mutant (Lab)	BTZ, DNB	Target Identification
M. bovis BCG Tokyo (Vaccine)	S	DST
M. smegmatis mc^2 155 (Lab)	S	DST
M. smegmatis mc^2 155, MN47 mutant (Lab)	BTZ, DNB	Target Identification
M. smegmatis mc^2 155, MN84 mutant (Lab)	BTZ, DNB	Target Identification

Phenotypes of each isolate are MDR (multidrug resistance), MDR+A (MDR+aminoglycoside), MDR+Q (MDR+ fluoroquinolone), XDR (extensive drug resistance), Rif (Rifampicin resistance), and S (Sensitive). BTZ: Benzothiazinone, DNB: dinitrobenzamide; DST : Drug susceptibility testing;
