# Supplementary material for: High Content Screening Identifies Decaprenyl-Phosphoribose 2′ Epimerase as a Target for Intracellular Antimycobacterial Inhibitors
Source: PLoS Pathog. 2009 Oct 30;5(10):e1000645. doi: 10.1371/journal.ppat.1000645 (PMC2763345; doi:10.1371/journal.ppat.1000645)
Supplement: Figure S3 — Dose-response analysis of Compounds 1 to 12-(S) listed in Table 2 in in vitro intracellular and in broth grown bacterial assays. Percentage of inhibition of intracellular growth from infected cells parameter (black squares) and extracellular growth (gray triangles) Results are shown as the mean of 2 independent experiments with standard deviation (SD). (0.09 MB PDF) [file ppat.1000645.s003.pdf]

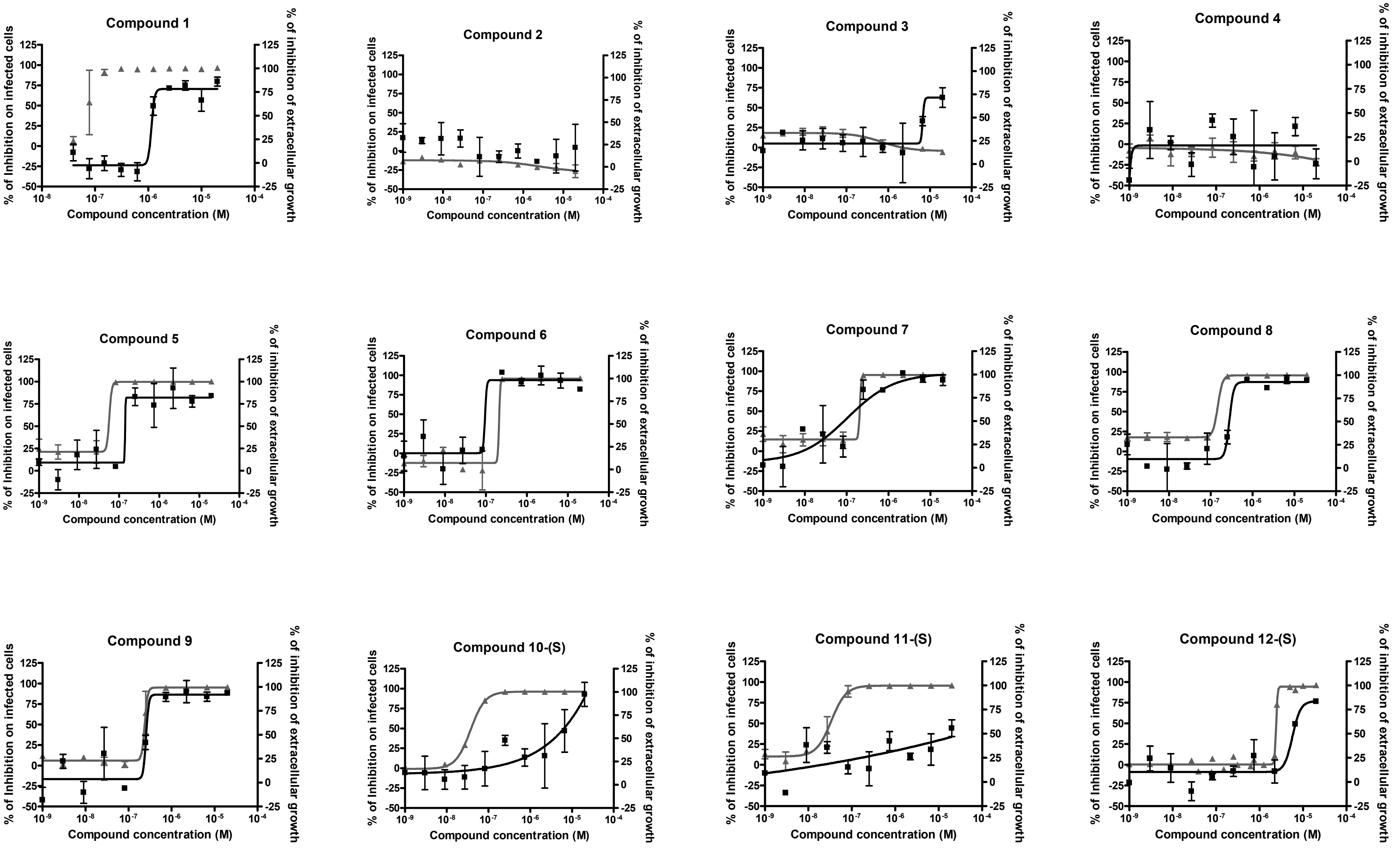
Compound 1
Compound 2
Compound 3
Compound 4
Compound 5
Compound 6
Compound 7
Compound 8
Compound 9
Compound 10-(S)
Compound 11-(S)
Compound 12-(S)
% of Inhibition on infected cells
% of inhibition of extracellular growth
Compound concentration (M)
